# Selectivity of Herbicides Applied Isolated and in Combinations in Grain Sorghum

**DOI:** 10.1021/acsomega.5c02778

**Published:** 2025-08-28

**Authors:** Bruno César Almeida, Denis Fernando Biffe, Jamil Constantin, Rubem Silvério de Oliveira Jr, Guilherme Braga Pereira Braz

**Affiliations:** † State University of Maringa, Maringa 87020-900, Parana, Brazil; ‡ 245072 Universidade de Rio Verde, Rio Verde 75901-970, Goias, Brazil

## Abstract

In Brazil, sorghum (*Sorghum bicolor*) has gained prominence as a second-crop option, serving as an alternative to maize, and is widely used both for straw production in no-until systems and for grain and forage for animal feed. However, weed management, particularly of grasses, within this crop is a significant challenge due to the limited availability of selective herbicides. Therefore, this study aimed to evaluate the selectivity of the herbicides trifluralin, atrazine, and mesotrione, applied individually or in combination during the postemergence phase of grain sorghum. Two field experiments were conducted to assess key variables including phytotoxicity, plant height, and grain yield. Applications of trifluralin and atrazine, either alone or in combination, resulted in mild to moderate phytotoxicity ranging from 5 to 16%, more pronounced at higher trifluralin rates, but did not negatively affect plant development or productivity. Similarly, the atrazine + mesotrione combination caused mild phytotoxicity symptoms, reaching 13%. In contrast, trifluralin + atrazine + mesotrione mixtures exhibited phytotoxicity levels ranging from 22 to 41% and led to significant productivity reductions across most evaluated dose combinations. These results highlight the importance of careful herbicide selection and appropriate application rates to achieve effective weed control without compromising the safety and productivity of sorghum crop.

## Graphical abstract

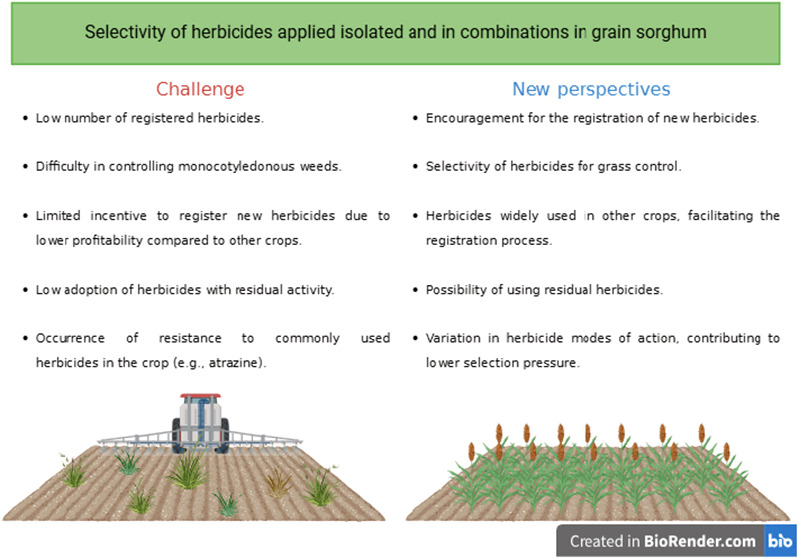

## Introduction

Sorghum (*Sorghum bicolor*) is a summer crop widely used in several countries such as United States, Nigeria, Sudan, India, Australia, and Argentina due to its high nutritional value, both for animal feed (forage and grains) and human consumption (grains).^1,2,3^ In Brazil, sorghum has gained prominence mainly as a second-season crop, serving as a viable alternative to replace corn, both for straw formation in no-tillage systems and for grain and forage production.^4–6^ Currently, one of the biggest challenges in successfully establishing the sorghum crop is the weed control, mainly the species classified as grasses, due to the crop’s sensitivity to grass-selective herbicides available in Brazil.^7^

When not properly controlled, weeds not only compete for water, light, space, and nutrients but also release allelopathic substances, interfere with harvesting, and serve as hosts for various insect pests, nematodes, and disease-causing pathogens.^8^ Weed species belonging to the same botanical family as the crop exhibit more intense competition for resources compared to species from different families due to their similarity in resource acquisition.^9^ It is estimated that weed competition with grain sorghum during the first 4 weeks after emergence can cause yield losses ranging from 40 to 97%.^10^ Grassy weed species pose greater concerns for the sorghum crop,^11^ exhibiting strong competitive ability with the crop even when present in lower densities compared to other species.^12^ The optimal period for weed control in grain sorghum is between the emergence of the fifth and ninth leaf.^13^

The differences in critical weed interference prevention periods reflect the distinct ecophysiological characteristics of different sorghum types, as well as variations in crop establishment and management across different seasons and locations, particularly regarding local soil and climate conditions, weed community composition, and infestation levels.^14^

It is important to highlight that some competing species exhibit morphology similar to the crop, which hinders selective control. In this context, the scarcity of selective herbicides for sorghum crop that provide control over grasses has led to the development of new technologies for this purpose. Currently, in Brazil, it is possible to use the herbicide S-metolachlor in the pre-emergence of sorghum crop, provided that the seeds are treated with a fluxofenim-based safener.^15^ Another recently introduced technology in the Brazilian market involves the cultivation of sorghum hybrids that exhibit tolerance to herbicides from the imidazolinone chemical group.^16^ The application of these acetolactate synthase (ALS) inhibitors can be carried out both in the pre-emergence and postemergence stages of the crop, as these hybrids are developed through conventional breeding.

The alternatives mentioned require the use of specific technologies to ensure their safety for the crop, such as safeners and genetic improvement. However, two other herbicides may assist in grass control and have demonstrated potential for use in this crop for this purpose, according to studies available in the literature, are mesotrione and trifluralin.^17,18^ Despite its importance as a grain-producing crop in the *Cerrado* region in Brazil, there are few studies on the selectivity of herbicides for this species.^19^ Given this gap and the need to establish efficient sorghum management techniques, it is essential to develop viable alternatives for weed control. This study aimed to evaluate the selectivity of herbicide combinations applied in the postemergence stage of grain sorghum.

## Material and Methods

Location and soil and climate conditions Two experiments were conducted in an experimental area located in the municipality of Mandaguaçu (PR), at the geographic coordinates of 23°13′33.97″S latitude and 52°01′14.57″W longitude, at an altitude of 464 m. The experimental period lasted from January 16, 2024, to May 14, 2024. Figure 1 presents the climatological data recorded during the experimental period.

**Figure 1:**
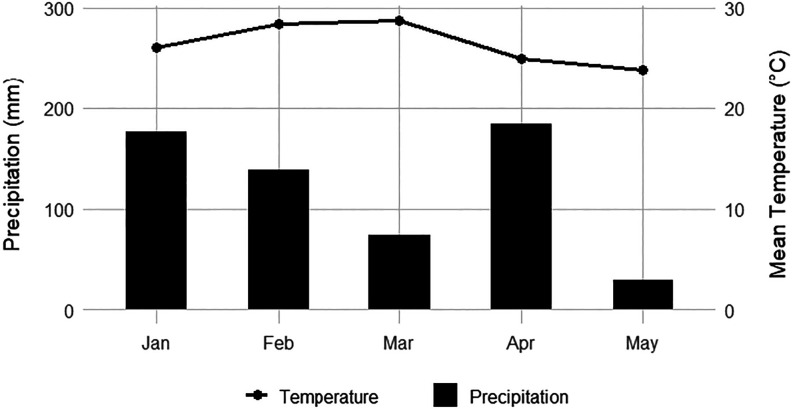
Climatological data during the experimental period. Source: Instituto Nacional de Meteorologia (INMET), Campus Headquarters Station, Universidade Estadual de Maringá (A835) – Maringá (Brazil).

The area is conducted in no-tillage system. The soil in the experimental area had a pH in water of 5.2; 3.08 cmolc of H^+^ + Al^3+^ dm^3^ of soil; 1.51 cmolc dm^3^ of Ca^2+^; 1.00 cmolc dm^3^ of Mg^2+^; 0.11 cmolc dm^3^ of K^+^; 52.31 mg dm^3^ of P; 8.26 g dm^3^ of C; and a texture composition of 78% sand, 2% silt, and 20% clay (medium-textured soil).

## Experimental Design and Description of Treatments

In both experiments, a randomized block design with an adjacent double-check scheme was used, with four replications. In this experimental arrangement, each evaluated treatment had two adjacent controls without herbicide application, which were used to compare the values obtained for each response variable. The adoption of this experimental design allows for the comparison (breakdown) of herbicide treatments with the adjacent controls installed within the same experimental unit, effectively minimizing area variability and experimental error. This is crucial for experiments evaluating herbicide selectivity.^20,21^ Figure 2 presents a graphical representation of the experimental design adopted in the study.

**Figure 2:**
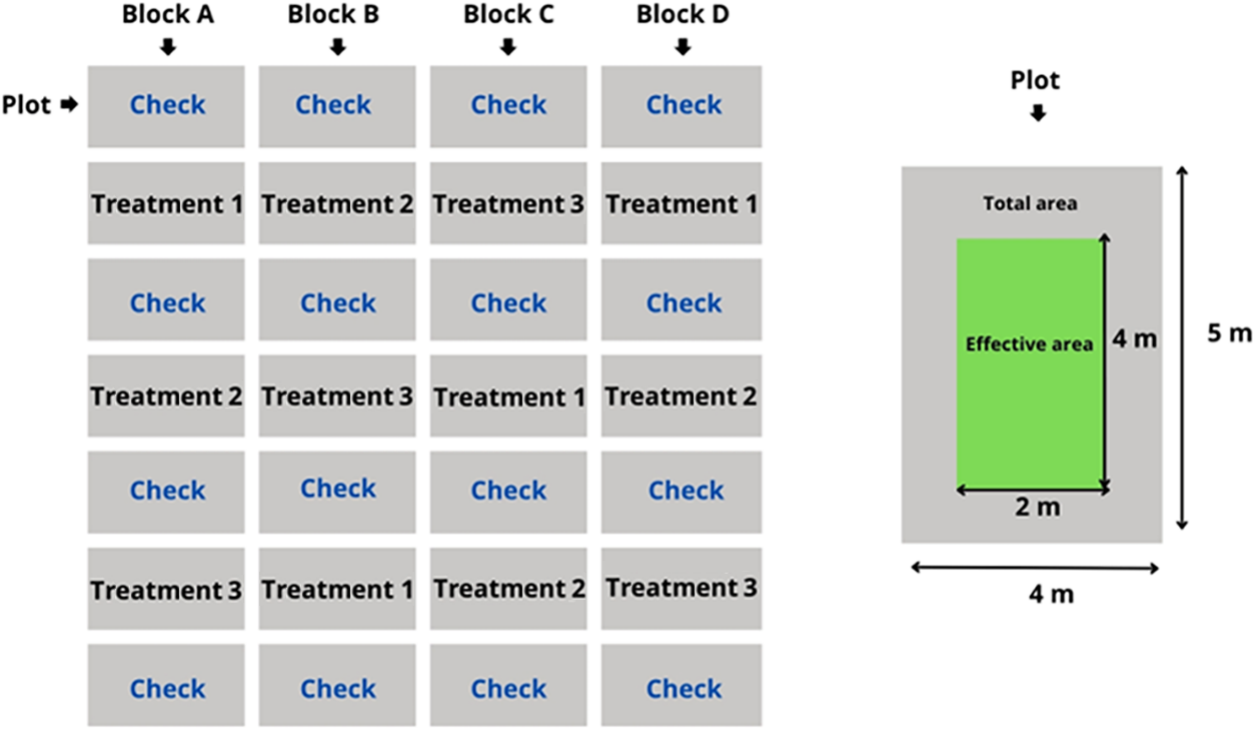
Schematic representation of the randomized complete block design with adjacent control used in the experiment. The diagram illustrates the spatial arrangement of treatments within the blocks, highlighting the plot dimensions, total area, and effective (usable) area. The figure serves as an example of the layout and does not depict all treatments.

For each experiment, the treatments consisted of the application of isolated or combined herbicides in the postemergence stage of sorghum. In Experiment 1, the evaluated treatments were: trifluralin (450; 675; 900; 1125; and 1350), trifluralin + atrazine (450 + 1000; 675 + 1000; 900 + 1000; 1125 + 1000; 1350 + 1000), and atrazine (1000). In Experiment 2, the evaluated treatments were trifluralin + atrazine + mesotrione (450 + 1000 + 43.2; 675 + 1000 + 43.2; 900 + 1000 + 43.2; 1125 + 1000 + 43.2; 1350 + 1000 + 43.2; 450 + 1000 + 72; 675 + 1000 + 72; 900 + 1000 + 72; 1125 + 1000 + 72; and 1350 + 1000 + 72) and atrazine + mesotrione (1000 + 43.2 and 1000 + 72). All the doses are expressed in g of active ingredient ha^–1^. The commercial products used were: trifluralin (Trifluralina Nortox Gold, EC, 450 g L^–1^, Nortox, Brazil), atrazine (Atrazina Nortox, SC, 500 g L^–1^, Nortox, Brazil), and mesotrione (Mesotriona Nortox, SC, 480 g L^–1^, Nortox, Brazil). The experimental units measured 4.0 m in width and 5.0 m in length, totaling a gross area of 20.0 m^2^. The effective area used for evaluations was limited to the central 2.0 m width and central 4.0 m length, totaling 8.0 m^2^.

## Crop Management and Response Variables Evaluated

The direct sowing of sorghum was carried out on January 15, 2024, using seeds of the NTXS 100 variety. Row spacing was 0.5 m, aiming to sow 9.8 seeds per linear meter at a depth of 3 cm. Fertilization consisted of 300 kg ha^–1^ of the 04–14–08 formula. Emergence occurred from January 20, 2024. Additionally, a top-dressing application of urea (80 kg ha^–1^) was performed on February 5, 2024. Throughout sorghum development, all crop management practices were carried out according to recommended guidelines, ensuring that pests and diseases did not interfere with crop development. Furthermore, weed control during the entire crop cycle was conducted manually to expose the plants solely to the effects of the postemergence herbicide treatments.

For all applications, a CO_2_-pressurized backpack sprayer was used, equipped with a boom containing four ST-110.015 flat fan nozzles spaced 0.50 m apart (application width of 2.0 m), operating at a pressure of 35 PSI. These application conditions resulted in a spray volume equivalent to 150 L ha^–1^. The treatments were applied on February 5, 2024, in both experiments, when sorghum plants were at the 3- to 4-leaf stage (third leaf with visible expanded ligule). At the time of application, soil moisture was adequate, the sky was cloud-free, and the minimum and maximum values for temperature, relative humidity, and wind speed were 27.1 and 29.2 °C, 61.0 and 65.3%, and 1.2 and 1.6 km h^–1^, respectively.

The evaluated variables were crop phytotoxicity at 14 and 28 days after application (DAA), assessed by the percentage of crop injury, where zero represents the absence of symptoms and 100% indicates complete plant death.^22^ Plant stand was evaluated by counting the number of plants in 4.0 m of the central rows. Average plant height was measured on 10 plants from the central row. Both evaluations were performed at 28 DAA.

Harvesting was conducted on May 14, 2024, when the number of panicles present in the effective area of the experimental units was counted. Grain yield was estimated by harvesting the panicles from the effective area of each experimental unit. After harvesting, the panicles were threshed, and a subsample from each plot was taken to determine grain moisture content using a *Mini GAC* portable moisture meter. Subsequently, grain yield was estimated in kilograms per hectare, with values corrected to 14% moisture content.

## Statistical Analysis

The statistical analysis of the experimental data was performed using SISVAR software.^23^ For phytotoxicity evaluations, data were subjected to analysis of variance, and when a significant effect was detected by the *F*-test (*p* ≤ 0.05), means were compared using the Scott-Knott test (*p* ≤ 0.05). For the other response variables, data from the herbicide-treated areas were compared to the mean of the adjacent double controls and subjected to analysis of variance using the *F*-test (*p* ≤ 0.05).

## Results and Discussion

### Experiment 1: Selectivity of Trifluralin Isolated and in Combination with Atrazine in Postemergence Application on Grain Sorghum

In the first phytotoxicity assessment, conducted at 14 DAA, levels ranged from 5.50 to 16.25% (Figure 3). The main symptom consisted of mild leaf margin curling in sorghum plants (Figure 4A). As higher doses of trifluralin were applied, regardless of whether it was combined with atrazine, increased intoxication levels in sorghum plants were observed. The absence of increased injury levels in sorghum plants due to the addition of atrazine in combination with trifluralin in postemergence applications can be explained by the fact that this herbicide is highly selective for the crop. Injuries are uncommon even at higher atrazine doses or at different application stages.^24,25^

**Figure 3:**
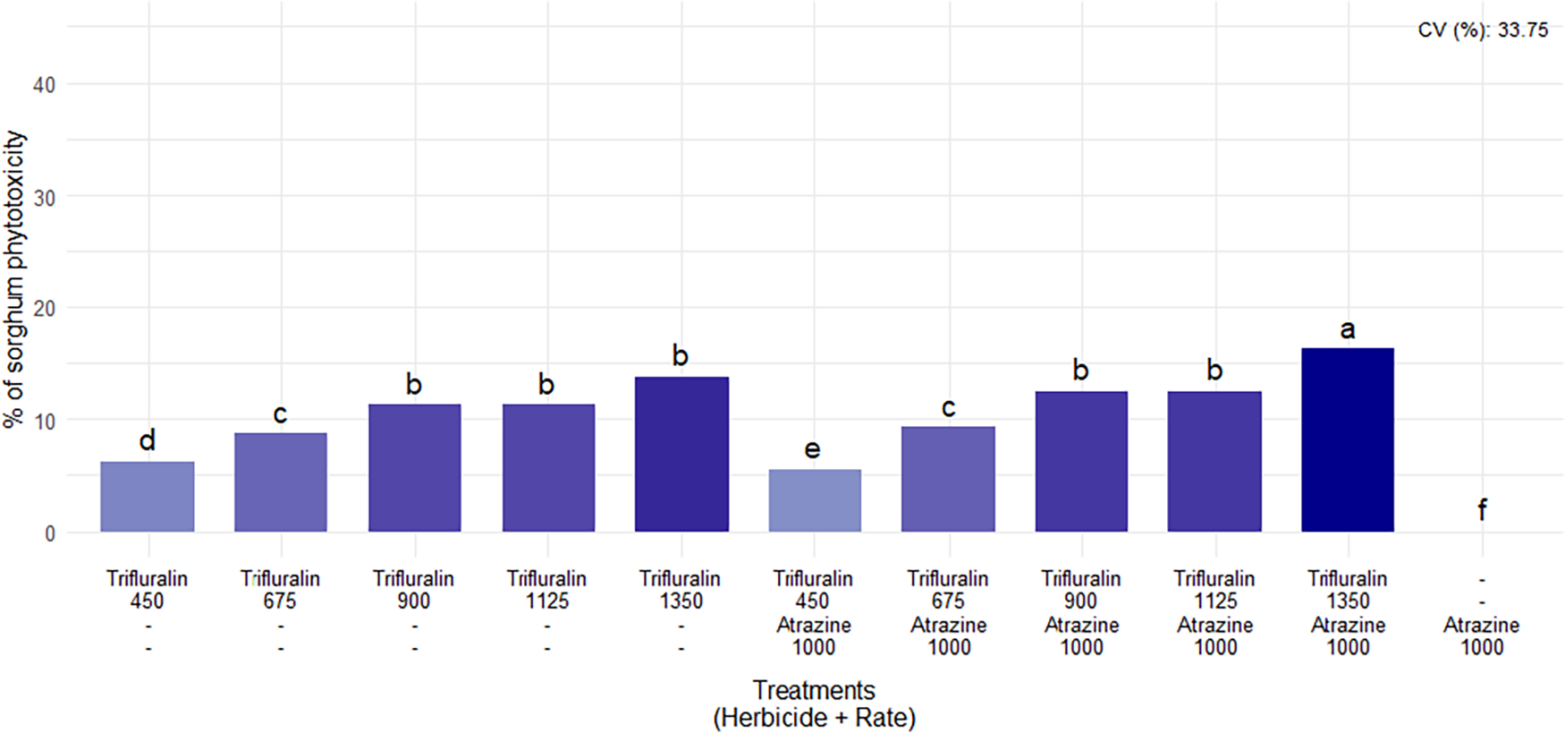
Phytotoxicity percentages of sorghum 14 DAA of postemergence herbicides. Herbicide doses are expressed in g a.i. ha^–1^. CV = coefficient of variation. Means followed by the same letter in the bar do not differ from each other according to the Scott-Knott test (*p* ≤ 0.05).

**Figure 4:**
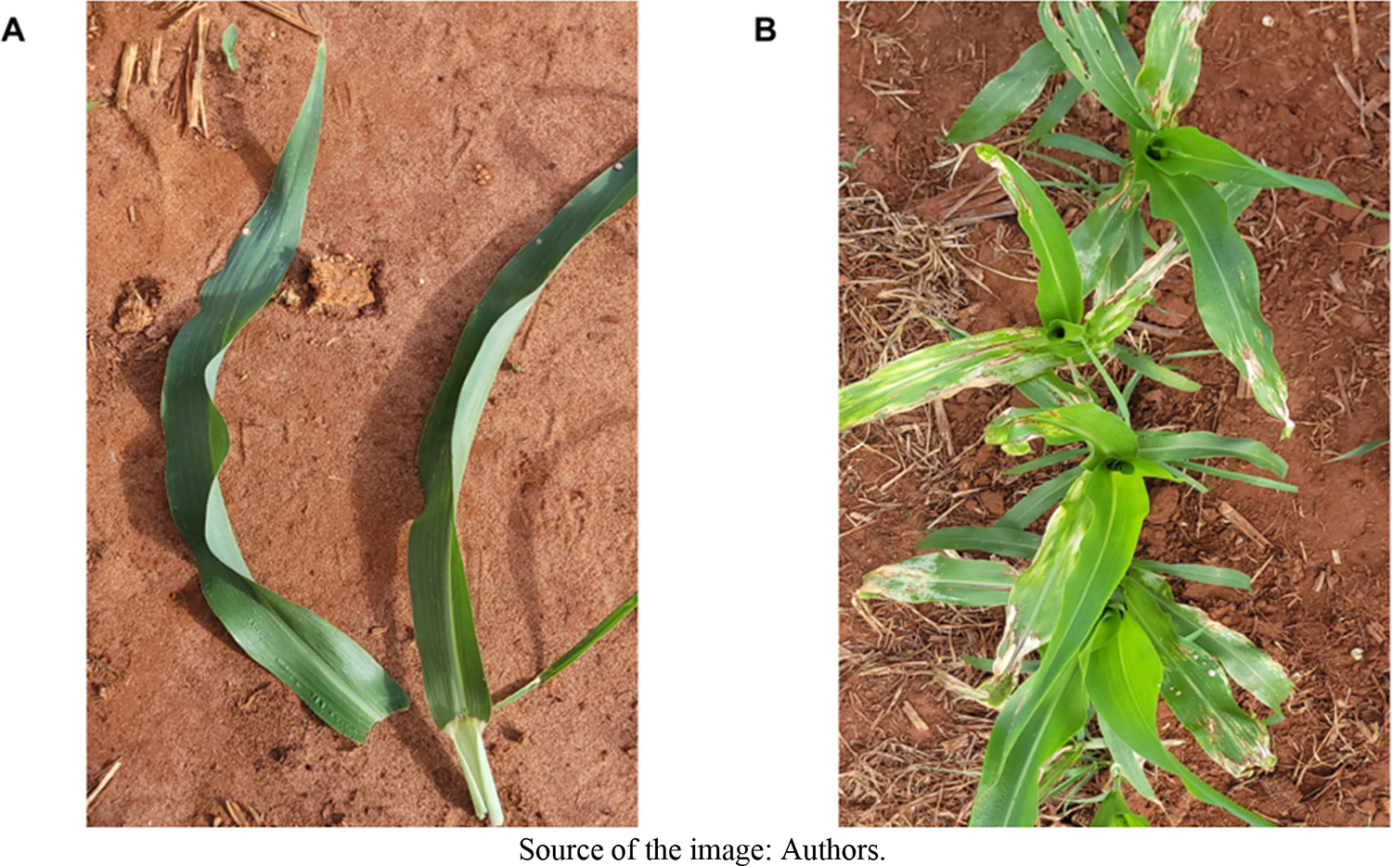
Visual symptoms of injuries caused by postemergence application in sorghum of the combination trifluralin + atrazine (A) and trifluralin + atrazine + mesotrione (B).

In the final phytotoxicity assessment, conducted at 28 DAA, no further injury symptoms were observed from the herbicides applied in postemergence on sorghum. This indicates the sorghum plants’ ability to recover from injuries caused by the use of trifluralin and atrazine, either isolated or in combination, when these active ingredients are applied in postemergence of the crop. Validating the selectivity of these herbicide combinations for sorghum, the key advantage lies in the ability to include an active ingredient with residual control spectrum (trifluralin) over grasses in postemergence applications of the crop.^26^

Table 1 presents the results for sorghum plant stand and height after the postemergence herbicide applications. For both response variables, none of the treatments caused reductions in plant density or height compared to their respective double controls, demonstrating that none of the herbicides, regardless of dose or whether applied isolated or in combination, caused harm to the sorghum crop for these parameters. As previously mentioned, atrazine’s selectivity for sorghum is well-documented, and it is the main herbicide used for weed control in this crop.

**Table 1:** Sorghum Plant Stand and Height at 28 DAA of Post-Emergence Herbicides Applied to the Crop^a^

		stand (plants in 4 m)			plant height (cm)		
treatments	doses (g ha^–1^)	TRT	DC	difference	TRT	DC	difference
trifluralin	450	21.25	25.50	–4.25	33.79	35.86	–2.07
trifluralin	675	25.25	27.25	–2.00	36.50	35.33	1.18
trifluralin	900	25.25	26.25	–1.00	35.57	33.04	2.54
trifluralin	1125	20.75	24.50	–3.75	35.79	34.57	1.21
trifluralin	1350	21.50	25.75	–4.25	35.25	34.61	0.65
trifluralin + atrazine	450 + 1000	22.75	19.25	3.50	33.28	35.39	–2.11
trifluralin + atrazine	675 + 1000	20.00	19.25	0.75	33.68	35.61	–1.93
trifluralin + atrazine	900 + 1000	20.25	20.50	–0.25	34.54	35.14	–0.61
trifluralin + atrazine	1125 + 1000	23.75	21.75	2.00	35.93	34.97	0.96
trifluralin + atrazine	1350 + 1000	22.50	20.00	2.50	33.79	37.00	–3.21
atrazine	1000	25.00	24.25	0.75	34.61	35.72	–1.11
CV (%)		2.66			16.47		
HSD		13.76			15.53		

a DAA = days after application; CV = coefficient of variation. HSD = honestly significant difference. TRT = Treatment; DC = Double check. Bold values indicate a statistical difference from the respective double check by the *F*-test (*p* ≤ 0.05).

The novelty of the results generated in this study lies in the absence of negative effects of trifluralin applied in postemergence on sorghum plant stand and height, with no influence of dose or combination with atrazine. This demonstrates that this active ingredient has the potential to be used in this application method for sorghum. The low potential of trifluralin to cause sorghum intoxication, as observed in this study, can be explained by its mode of action. Since it acts by inhibiting microtubule formation, affecting cell division in growing plants, its most pronounced effects are typically seen in seeds undergoing germination or in newly emerged seedlings.^27^ In this study, trifluralin was applied in postemergence at a phenological stage where the damage caused by this herbicide was mitigated by the significant vegetative mass of the plants at the time of application.

To assess the effect of herbicide treatments on sorghum yield parameters, evaluations of the number of panicles and grain yield were conducted (Figures 5 and 6). Similar to what was observed for plant stand and height, no negative effects of herbicide treatments were detected for either the number of panicles or grain yield compared to their respective double controls. More specifically, postemergence application of trifluralin isolated or in combination with atrazine, regardless of the dose used, did not result in a decrease in the number of sorghum panicles nor did it lead to reductions in grain yield.

**Figure 5:**
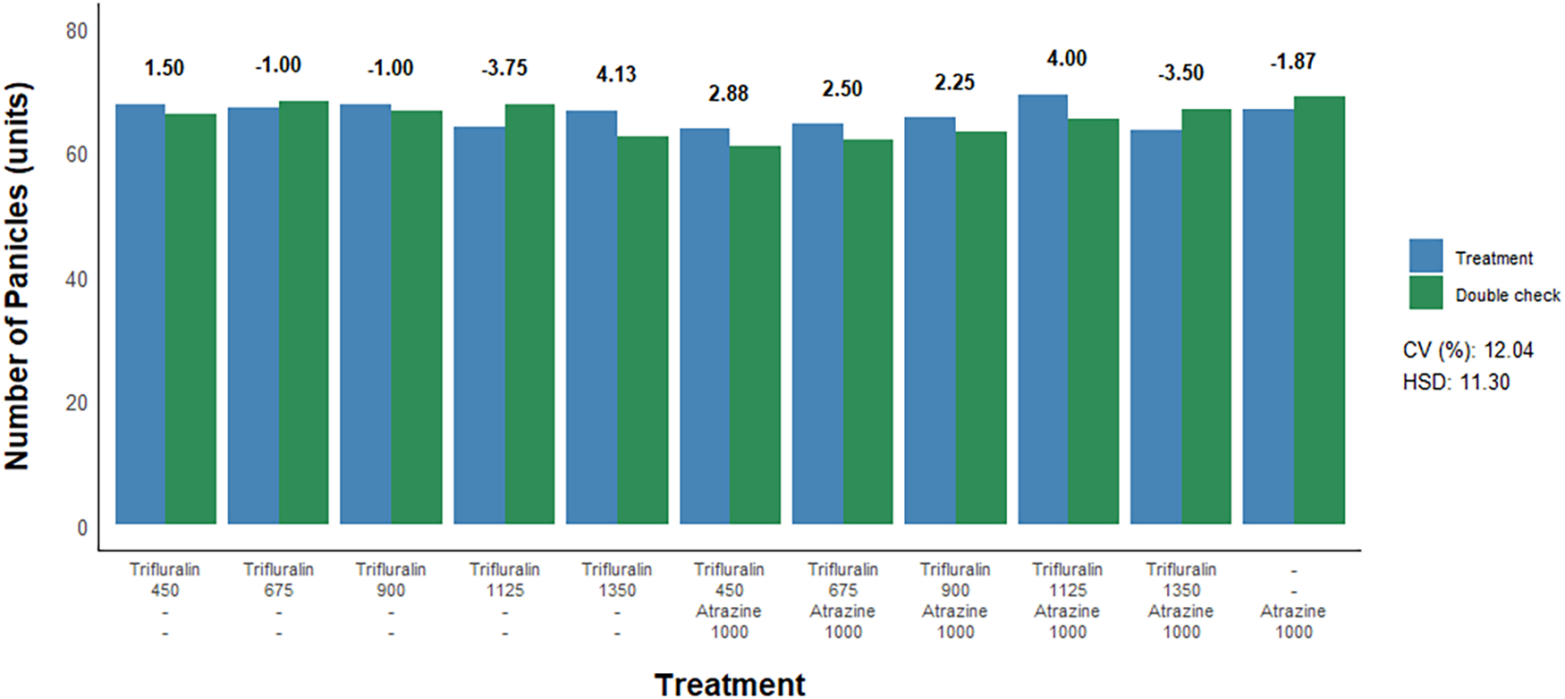
Number of panicles of sorghum as a function of postemergence herbicide application to the crop. Herbicide doses are expressed in g a.i. ha^–1^. CV = coefficient of variation. HSD = honestly significant difference. Bold red values indicate a statistical difference from the respective double check by the *F*-test (*p* ≤ 0.05).

**Figure 6:**
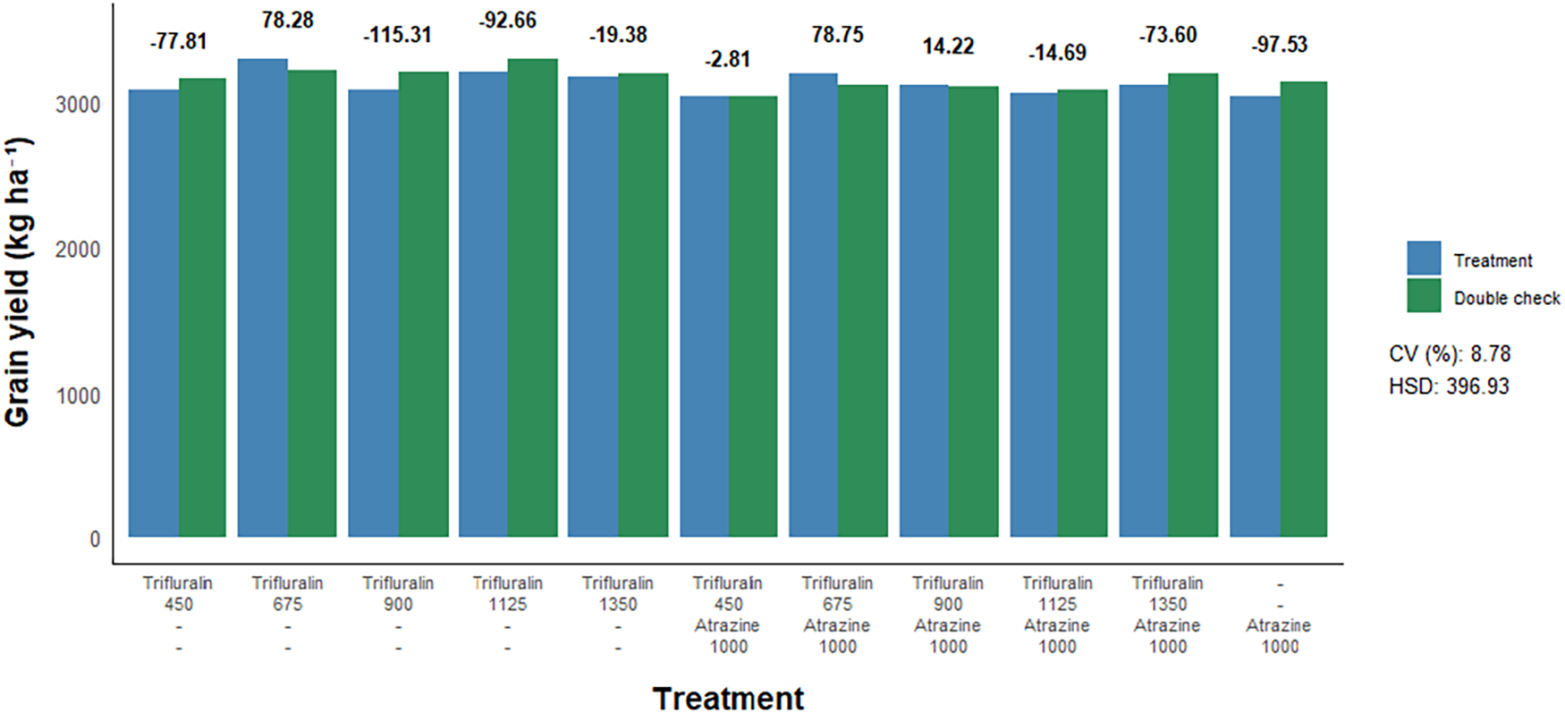
Grain yield of sorghum as a function of postemergence herbicide application to the crop. Herbicide doses are expressed in g a.i. ha^–1^. CV = coefficient of variation. HSD = honestly significant difference. Bold red values indicate a statistical difference from the respective double check by the *F*-test (*p* ≤ 0.05).

The fact that trifluralin demonstrated selectivity within the evaluated dose range when applied in postemergence creates an opportunity to expand chemical weed control strategies in sorghum, given the currently limited availability of selective herbicides for this crop. Additionally, it opens the possibility of implementing chemical control systems for grasses, which are among the most problematic weeds in sorghum. Due to the botanical family similarity (Poaceae) between these weed species and the crop, there are greater restrictions on effective and selective herbicides.

In this context, one possible grass weed control system in sorghum involves the use of S-metolachlor in pre-emergence, with seed treatment using the safener fluxofenim.^15^ This pre-emergence application could be complemented by a postemergence application of trifluralin + atrazine, which would extend residual weed control throughout the crop cycle, reduce the weed seed bank in highly infested areas, and enable clean harvesting. However, since this approach has not yet been validated across a wide range of conditions, further studies are recommended. These studies could determine whether edaphoclimatic conditions, crop growth stage at the time of application, or differential tolerance among sorghum hybrids influence herbicide selectivity, as these factors have been critical in confirming selectivity for other active ingredients in sorghum.^25,28^

### Experiment 2: Selectivity of the Combination of Trifluralin + Atrazine + Mesotrione Applied in Postemergence of Grain Sorghum

In the first phytotoxicity assessment (14 DAA), regardless of the herbicide combination applied in postemergence of sorghum, injuries were observed in plants subjected to all treatments (Figure 7). The symptoms were characterized by chlorotic spots along the leaf blade and slight albinism in the younger plant tissues (Figure 8B). These symptoms are associated with the addition of mesotrione in the herbicide combinations. Due to the mode of action of this active ingredient, it is common to observe chlorosis in already developed leaf tissues and foliar bleaching (albinism) in newly formed tissues, followed by tissue necrosis caused by free radical-induced damage to cell membranes.^29^

**Figure 7:**
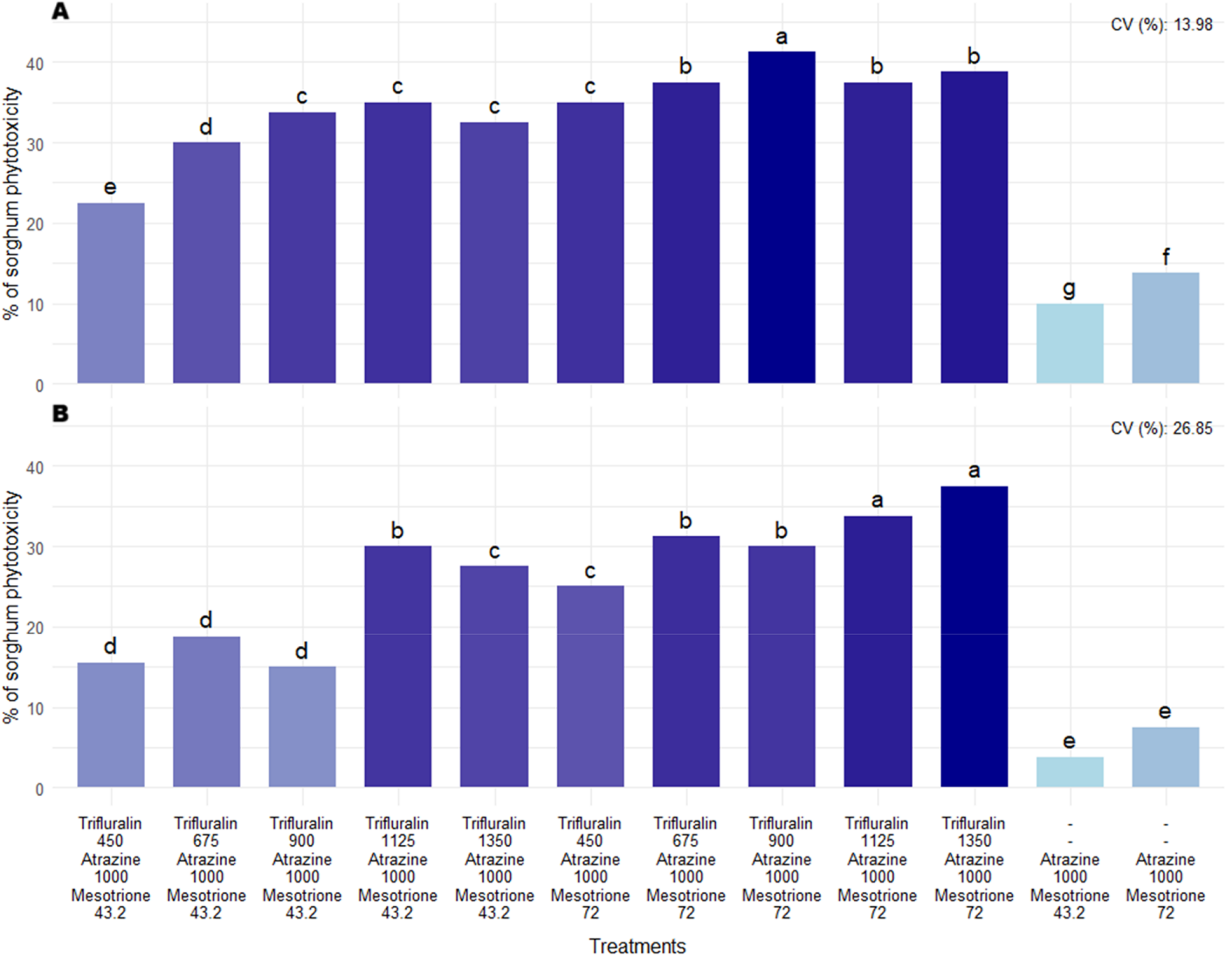
Phytotoxicity percentages of sorghum in evaluations conducted after the application of postemergence herbicides. (A) Corresponds to the 14 days after application (DAA) assessment, and, (B) to the 28 DAA assessment. Herbicide doses are expressed in g a.i. ha^–1^. CV = coefficient of variation. Means followed by the same letter in the bar do not differ from each other according to the Scott-Knott test (*p* ≤ 0.05).

**Figure 8:**
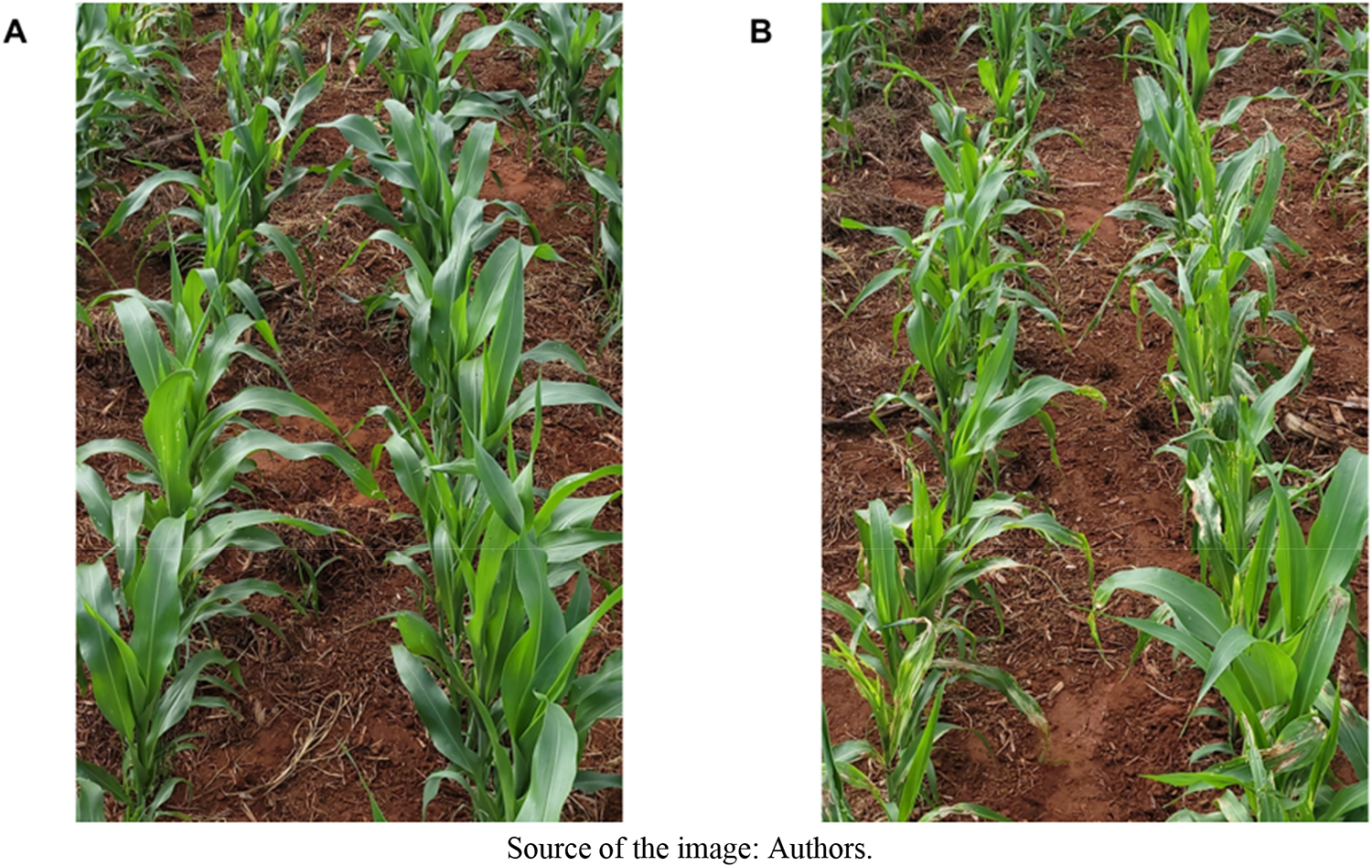
Visual symptoms of phytotoxicity in sorghum: (A) no herbicide application (check), and (B) postemergence treatment with trifluralin + atrazine + mesotrione.

In the assessment conducted at 14 DAA, phytotoxicity levels were high in all treatments where the triple combination of trifluralin + atrazine + mesotrione was used, with injuries observed in proportion to the mesotrione dose applied in the treatments (Figures 7A and 8B). For treatments consisting of atrazine + mesotrione, the levels of sorghum plant intoxication were lower compared to those where the triple herbicide combination was applied. Although there are currently no mesotrione-based products registered for use in sorghum in Brazil, several studies have already demonstrated the selective potential of this herbicide for postemergence applications in this crop,^24,30,31^ and mesotrione-based products are registered for use in other countries. In a follow-up assessment conducted at 28 DAA, a reduction in the level of injuries observed in sorghum plants was noted, although high levels of phytotoxicity persisted in some treatments, especially those in which the triple combination with the highest mesotrione dose (72 g ha^–1^) was applied.

Although the injury levels caused by the postemergence herbicide applications in sorghum were high, no plant mortality was observed, as there were no differences in crop stand when comparing the treatments with their respective double controls (Table 2). On the other hand, regarding plant height, it was observed that in the treatment with the application of the trifluralin + atrazine + mesotrione combination (1125 + 1000 + 72 g ha^–1^), there was a reduction in the growth rate, resulting in shorter plants compared to the respective double check of this treatment. For all other treatments, no differences were observed in the height of sorghum plants that received postemergence herbicide applications compared to the controls.

**Table 2:** Sorghum Plant Stand and Height at 28 DAA of Post-Emergence Herbicides Applied to the Crop^a^

		stand (plants in 4 m)			plant height (cm)		
treatments	doses (g ha^–1^)	TRT	DC	difference	TRT	DC	difference
trifluralin + atrazine + mesotrione	450 + 1000 + 43.2	24.50	25.00	–0.50	32.57	33.72	–1.15
trifluralin + atrazine + mesotrione	675 + 1000 + 43.2	21.75	22.00	–0.25	28.86	33.75	–4.89
trifluralin + atrazine + mesotrione	900 + 1000 + 43.2	22.50	24.00	–1.50	31.33	33.07	–1.75
trifluralin + atrazine + mesotrione	1125 + 1000 + 43.2	21.75	21.00	0.75	30.68	34.14	–3.46
trifluralin + atrazine + mesotrione	1350 + 1000 + 43.2	17.50	23.25	–5.75	29.08	32.86	–3.78
trifluralin + atrazine + mesotrione	450 + 1000 + 72	24.00	22.00	2.00	32.39	32.43	–0.04
trifluralin + atrazine + mesotrione	675 + 1000 + 72	21.75	22.25	–0.50	31.04	36.14	–5.11
trifluralin + atrazine + mesotrione	900 + 1000 + 72	22.50	21.75	0.75	32.68	35.25	–2.57
trifluralin + atrazine + mesotrione	1125 + 1000 + 72	21.50	22.75	–1.25	29.00	35.90	–6.90
trifluralin + atrazine + mesotrione	1350 + 1000 + 72	23.50	21.50	2.00	28.86	34.39	–5.53
atrazine + mesotrione	1000 + 43.2	21.00	22.00	–1.00	35.04	36.79	–1.75
atrazine + mesotrione	1000 + 72	21.75	22.75	–1.00	32.39	34.46	–2.07
CV (%)		20.22			7.31		
HSD		12.04			6.45		

a DAA = days after application; CV = coefficient of variation. HSD = honestly significant difference. TRT = Treatment; DC = Double check. Bold values indicate a statistical difference from the respective double check by the *F*-test (*p* ≤ 0.05).

Figures 9 and 10, presents the data on panicle number and grain yield evaluations. Regarding the yield component panicle number, no negative effects were observed from the postemergence herbicide treatments in sorghum compared to their respective double controls. However, sorghum grain yield was affected in eight out of the 12 herbicide combinations evaluated, compared to their respective double checks.

**Figure 9:**
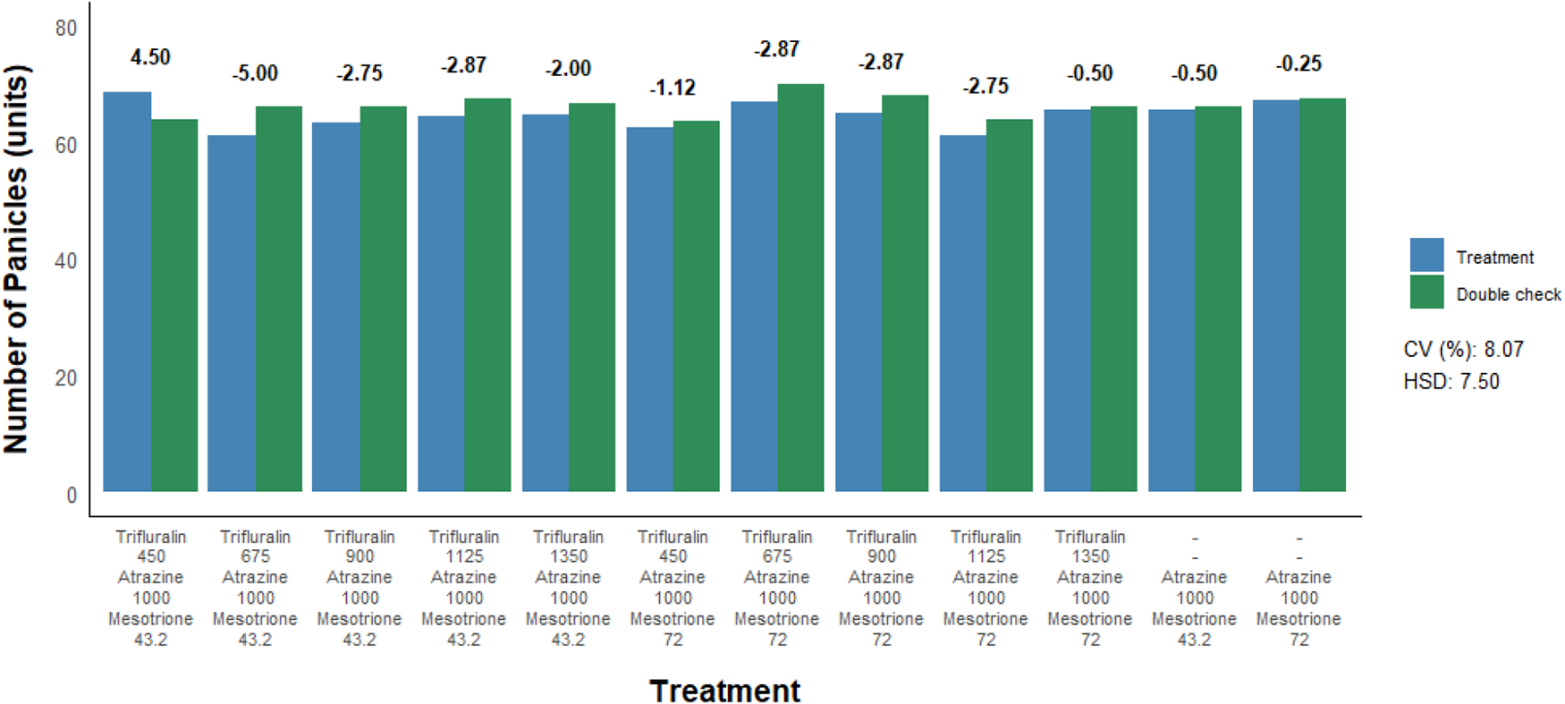
Number of panicles of sorghum as a function of postemergence herbicide application to the crop. Herbicide doses are expressed in g a.i. ha^–1^. CV = coefficient of variation. HSD = honestly significant difference. Bold red values indicate a statistical difference from the respective double check by the *F*-test (*p* ≤ 0.05).

**Figure 10:**
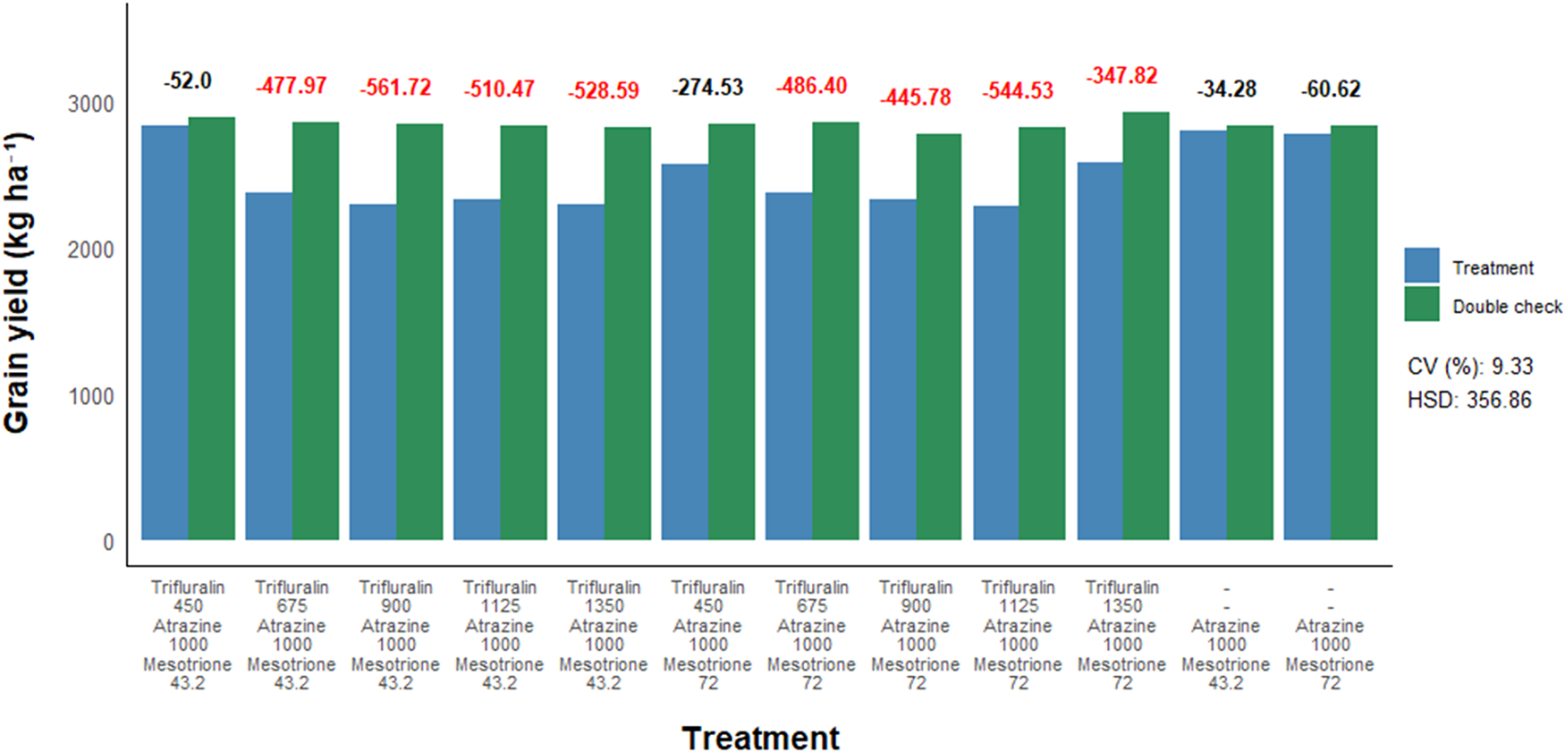
Grain yield of sorghum as a function of postemergence herbicide application to the crop. Herbicide doses are expressed in g a.i. ha^–1^. CV = coefficient of variation. HSD = honestly significant difference. Bold red values indicate a statistical difference from the respective double check by the *F*-test (*p* ≤ 0.05).

Within the group of treatments containing the triple herbicide combination, only those in which trifluralin was applied at the lowest evaluated dose (450 g ha^–1^) did not affect sorghum yield compared to their respective double controls. The two treatments consisting of the atrazine + mesotrione combination, regardless of dose, also did not affect sorghum grain yield. In this sense, only the treatments composed of the atrazine + mesotrione combination (1000 + 43.2 or 1000 + 72 g ha^–1^) and the triple combination trifluralin + atrazine + mesotrione (450 + 1000 + 43.2 or 450 + 1000 + 72 g ha^–1^) did not affect sorghum yield when applied in postemergence.

## Conclusions

Postemergence application of the triple mixture of trifluralin + atrazine + mesotrione in sorghum resulted in high levels of phytotoxicity, 41,25%. However, the triple mixture treatments did not affect plant stand or panicle number, but did reduce yield at most evaluated rates, especially at higher doses of trifluralin and mesotrione, indicating a lack of safety for use in the crop.

For the combination of atrazine and mesotrione, at both evaluated dose comparisons, injury levels observed in sorghum plants were mild.

Treatments containing trifluralin, either alone or in combination with atrazine, caused mild to moderate phytotoxicity symptoms in between 5 and 16%., with more severe injuries at higher trifluralin doses. However, plant recovery was observed from 28 days after application. Additionally, postemergence application of trifluralin, regardless of dose or combination with atrazine, did not negatively affect plant stand, height, panicle number, or grain yield when compared to the respective double controls, demonstrating the selectivity of these treatments for the crop.

Thus, producers facing difficulties in controlling grasses in sorghum fields may safely use trifluralin alone or in combination with atrazine, without compromising crop yield.

## Abbreviations

DAA: days after application
TRT: treatment
DC: double check
